# Antibacterial Metallic Touch Surfaces

**DOI:** 10.3390/ma9090736

**Published:** 2016-08-29

**Authors:** Victor M. Villapún, Lynn G. Dover, Andrew Cross, Sergio González

**Affiliations:** 1 Faculty of Engineering and Environment, Northumbria University, Newcastle upon Tyne NE1 8ST, UK; victor.puzas@northumbria.ac.uk; 2 Department of Applied Sciences, Faculty of Health and Life Sciences, Northumbria University, Newcastle upon Tyne NE1 8ST, UK; lynn.dover@northumbria.ac.uk; 3 ACT Surfaces Ltd., 1 Bearcroft Avenue, Worcester WR4 0DR, UK; andrew@act-surfaces.co.uk

**Keywords:** copper, disinfection, antimicrobial, touch surfaces

## Abstract

Our aim is to present a comprehensive review of the development of modern antibacterial metallic materials as touch surfaces in healthcare settings. Initially we compare Japanese, European and US standards for the assessment of antimicrobial activity. The variations in methodologies defined in these standards are highlighted. Our review will also cover the most relevant factors that define the antimicrobial performance of metals, namely, the effect of humidity, material geometry, chemistry, physical properties and oxidation of the material. The state of the art in contact-killing materials will be described. Finally, the effect of cleaning products, including disinfectants, on the antimicrobial performance, either by direct contact or by altering the touch surface chemistry on which the microbes attach, will be discussed. We offer our outlook, identifying research areas that require further development and an overview of potential future directions of this exciting field.

## 1. Introduction

Despite great investment in resources devoted to environmental and personal hygiene, rates of nosocomial or hospital-acquired infections (HAI) remain significant. A recent European survey [1] estimated that the total annual number of patients with HAI in European acute care hospitals was 3.2 million in 2011–2012. Another important issue is the continued emergence of antibiotic resistances in healthcare, which is a growing concern for public health. However, great variation is seen in the incidence of antimicrobial resistance across Europe [2].

To tackle this problem, new antimicrobial materials have been developed. Metallic alloys have been widely used since ancient times to limit microbial activity. For example, silver bottles and vessels were used by Phoenicians, Romans and Greeks to store water to prevent it being spoiled by bacteria. Copper was widely used in the pre-antibiotic era to tackle a plethora of infections ranging from tuberculosis to skin problems [3]. To implement their use in the healthcare sector, these antimicrobial elements could be mixed with other metals to optimise the final properties of the resulting alloy. For example, brasses are a mixture of copper and zinc that could be used in hospital door furniture such as push plates and doorknobs due to their antimicrobial activity. The hardness, antimicrobial performance and colour of the alloy can be tuned by changing the copper-to-zinc ratio. Other mixtures such as copper-silver and copper-nickel, enable improvements in the anticorrosion performance and durability of copper while maintaining good antimicrobial activity [4]; however, copper-silver is primarily used for electrical applications while nickel-containing alloys are not recommended for touch surfaces since nickel is a metal allergen.

Now, with antibiotic resistance on the rise, including the emergence of multi-drug resistant superbugs able to tolerate the last-resort antibiotic (colistin), a renewed drive to develop antimicrobial materials is needed urgently. Hospital touch surfaces such as bed rails and handles can become a reservoir of pathogens that cause patient infections resulting not only in substantial economic losses, but even in patient death. Traditionally, stainless steel is used and sterilised on a regular basis. However, researchers have realised that this approach may not effectively prevent the transmission of bacteria due to the large number of surfaces in hospitals and the fact that their sterilisation is transient as the materials themselves do not possess inherent antimicrobial activity. Nosocomial infections are therefore a real hazard, and using antimicrobial touch surfaces could be useful against pathogenic microbes. Among these materials, copper is the most frequently used [5] due to its efficiency in “contact killing”; however, silver and zinc oxides also exhibit antimicrobial activity, which are generally used in the form of nanoparticles. The antimicrobial properties have been generally attributed to the release of ions from their surface, the rate of which mainly depends on the chemical composition of the material [6,7,8].

**Figure 1:**
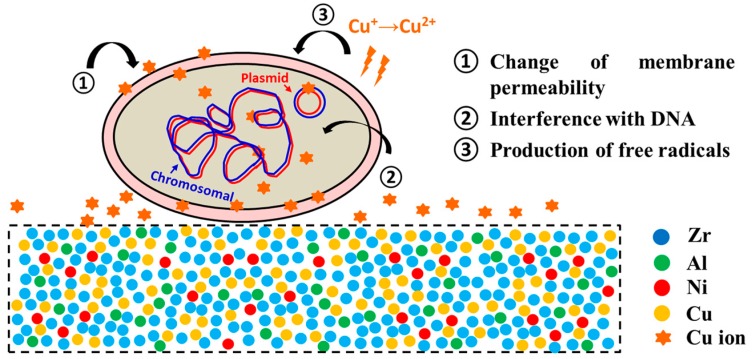
Representation of the contributing antimicrobial mechanisms of Cu ions released by a Cu bearing Zr-based bulk metallic glass [11].

The disruption of microbes due to the presence of Cu ions appears to occur in three parallel ways (Figure 1) and as such, prevents the risk of bacterial resistance. Contact with copper surfaces has been shown to damage the integrity of bacterial membranes, Cu ions can directly damage bacterial proteins and they can also induce the formation of highly damaging hydroxyl radicals via a Fenton-like chemistry, which can then damage the cells via their interactions with DNA, enzymes and other proteins, as well as the peroxidation of lipids and subsequent membrane damage [9,10,11].

Despite the growing interest in the topic, there are still many questions that remain open such as the key factors affecting antimicrobial performance. Moreover, reviews and manuscripts dealing with the topic are very scarce (e.g., one of the reviews was recently published by Vincent et al. [12]) and therefore we aim to fill the gap by providing a critical overview of the topic and to highlight future potential developments and trends in the field. This review will cover important aspects of the standards for antimicrobial assessment and the need for microbiological benchmarks. Some of the most important factors responsible for the antimicrobial behaviour such as the humidity, composition and oxidation conditions will be critically discussed. Additionally, we will consider the effects of cleaning products and disinfectants, an issue of the utmost importance that has been mostly overlooked elsewhere.

## 2. Standards for Antimicrobial Assessment

There are three major international standards used for the assessment of antimicrobial activity of surfaces; the major features of the Japanese, European and a newly proposed US standard are summarised in Table 1. The proposed US EPA protocol is the most specifically directed toward copper surfaces. The three standards have been developed using the pathogens *Staphylococcus aureus* and either *Escherichia coli* or *Pseudomonas aeruginosa*. *S. aureus* is a common coloniser of the human nasopharynx and skin. Often without symptoms, it also causes a broad range of diseases and is particularly adept at gaining entry to patients via in-dwelling medical devices and wounds, including surgical sites [13].

Particularly concerning is methicillin-resistant *S. aureus* (MRSA), which is widespread and has broad resistance to β-lactam antibiotics [13]. Many strains of *E. coli* are harmless organisms that inhabit our intestines but there are several pathotypes that cause intestinal disease (both haemolytic and non-haemolytic diarrhoeas via a variety of mechanisms), and infections of the urinary tract, bloodstream and central nervous system [14]. Furthermore, these bacteria are well-studied exemplars of two key bacterial archetypes, the Gram-positive (*S. aureus*) and Gram-negative (*E. coli*) bacteria. In place of *E. coli*, the US EPA protocol uses *P. aeruginosa*, another Gram-negative organism that is found widely in the environment. *P. aeruginosa* has intrinsic resistance to a range of antibiotics, as well as causes infections of wounds and burns and progressive infections. It also colonises the lungs of patients with congestive lung disorders such as cystic fibrosis, bronchiecstasis and chronic obstructive pulmonary disorder causing inflammation and ultimately progressive loss of lung function [15].

JIS Z2801:2010 and ISO 22196:2011 define a methodology for measuring antimicrobial performance in a variety of materials and define how this should be calculated and expressed. Essentially, a standardised inoculum of 6 × 10^5^ cells/mL is applied to antimicrobial test pieces (40 mm × 40 mm) and otherwise identical samples that lack the active component. The bacteria are incubated for a fixed period of 24 ± 1 h at 35 ± 1 °C with high relative humidity (>90%). The recovery of the inoculum and 24 h survivors is quantified by dilution in broth medium and subsequent counting of colonies that develop on agar plates. There are validation criteria relating to reproducibility of technical replicates and adequate recovery of bacteria from control (non-microbicidal) surfaces.

The United States Environmental Protection Agency (US EPA) [16] have recently developed a protocol for the evaluation of bactericidal activity of hard and non-porous copper/copper-alloy surfaces that is currently being considered for adoption in a consultation process. The antimicrobial parameters of this protocol are similar to the other standards but several differences are evident. Firstly, the EPA protocol not only provides the equations and conditions to develop antimicrobial tests but also provides normalised methods to assess the impact of biocidal cleaning liquids (Sodium hypochlorite, Hydrogen peroxide and Phosphoric acid) on non-porous surfaces. Disinfection by biocidal liquids is a daily procedure for materials meant to be used for their bactericidal properties. The EPA protocol provides a means to assess the changes in mechanical and chemical properties of this process in copper-based materials [16].

The US standard clearly states that a material can be considered as a sanitizer only when greater than 99.9% of these pathogens are eliminated within 1 h. The Japanese and European standards do not provide an antibacterial activity threshold but provide a framework for standardised quantification of antimicrobial activity; the range of products they cover are somewhat broader and thus benchmarking is more difficult to develop.

The number of anti-microbiological studies that are performed following the standards are very scarce. For example, Chu et al. [17] proposed the use of the Japanese Industrial Standard JIS Z2801:2000 to assess the antimicrobial properties of Zr-Cu-based thin films. The value of the antimicrobial activity proposed by this standard is calculated through the formula *R* = log (*B*/*A*) where *R* is the antimicrobial activity, *A* is the average number of viable cells of bacteria after 24 h inoculation of the treated test piece and *B* is the average of the number of viable cells of bacteria on the untreated test piece after 24 h. Nevertheless, the authors decided to calculate the antimicrobial rate as $\frac{N_{o}-N}{N}\times 100\%$ where *N*_0_ and *N* are the number of viable microbes on a control sample and on the prepared thin film respectively [17]. The same equation was used by Sharifahmadian et al. to compare the antimicrobial rate of wire arc spray copper coatings with that of commercial copper and stainless steel [18].

The antimicrobial activity of copper is characterised by a high bactericidal rate. Test conditions based on 24 h of interaction are not able to completely characterise a touching surface. As a practical example, doorknobs or door plates in hospitals are continuously touched. In these cases, the transmission of pathogen caused by touching surfaces may take place in less than 24 h. This means that even if 99.99% of bacteria are eliminated after 24 h, the transmission of pathogen may take place. For this reason, the antibacterial performance of materials needs to be analysed over shorter periods. As far as the authors are concerned, this inconsistency may be caused by the lack of an international standard which normalises antimicrobial activity tests for touching surfaces. In addition, a benchmark time that can be used to evaluate when nosocomial infection is prevented seems to be lacking. Some researchers have recently proposed 5 min as the optimum time that bacteria should be eliminated from touch surfaces in order to limit transmission [19]. This was considered the average estimated time for two people to touch the same surface in hospitals. A suitable test of antibacterial activity should be proposed based upon the application of a standardised bacterial inoculum at material surfaces over an authentic time course.

## 3. Factors Responsible for Antimicrobial Behaviour

### 3.1. Adhesion of Bacteria to Antimicrobial Surfaces

The antimicrobial performance of a material depends on a variety of parameters but the adhesion of bacteria to surfaces plays a major role. Several researchers use assays that essentially measure the antimicrobial effect of solid materials by their ability to kill or inhibit the growth of bacteria at some distance from the surface, for example in the application of surfaces to agar plates or the immersion of materials in broth cultures. The major factors measured in these systems are the ability of the material to produce ions which diffuse and influence the growth of bacteria in the surrounding culture medium. These are particularly suitable for applications such as emulating the flow of water through pipes but more authentic methods for touch surfaces need to limit the physical distance between the bacteria and the substrate surface. The standardised tests described in the previous section are more appropriate in this regard, since bacteria in close proximity to the ion-generating surface will be subject to the greatest antibacterial activity.

The process by which bacteria adhere to a surface is somewhat complex and is dominated by their surface chemistry. It essentially occurs in two steps [20]. Initially, the movement of bacteria from an aqueous phase to a material surface is influenced by their ability to move (motility) or be moved by Brownian motion and/or gravity and, when in closer proximity to the surface (<5 nm), they are attracted to the surface by short-range forces such as electrostatic charge, van der Waals attraction and hydrophobic interactions. On many surfaces, especially human tissues or the surfaces of medical implants, this initial adherence facilitates more specific molecular interactions between bacteria and surfaces. In order to more thoroughly understand the factors and forces that determine bacterial adhesion to surfaces, some researchers have considered whether bacterial attachment to surfaces is governed by the same physicochemical interactions that determine deposition of non-living colloidal particles [20].

It is clear that a number of different factors affect bacterial adhesion. Environmental factors such as the number of bacteria present, the ambient temperature, the duration of exposure, pH and the presence of disinfectants will all influence adhesion. Likewise, by changing the surface chemical composition it is possible to tune the charge and hydrophobicity of the material and therefore to control the adhesion and subsequent proliferation of bacteria. An important factor is that touch surfaces are subject to the build-up of organic deposits derived from the exudations of human skin and therefore result in very different adhesion properties to the metal surface itself and will serve to support bacterial adherence. Moreover, the ability of bacteria to adhere to each other and to develop a complex community at a surface as a biofilm should not be underestimated. These biofilms are protected from environmental challenge and physical removal by a polysaccharide matrix. Dental plaque is a useful example of a biofilm which can form on metal surfaces used in orthodontics and dental surgery [21,22,23].

The topology of the surface itself is another important factor. Rough (unpolished) surfaces bear surface irregularities that promote bacterial adhesion and biofilm deposition while very smooth (polished surfaces) do not favour the adhesion and deposition [24]. Beyond these visibly obvious features, surface configuration is also important: bacteria adhere and colonise porous surfaces preferentially over dense materials. Microscopic scratches or grooves of a similar size to the bacteria serve to increase the contact area available for adhesion and therefore the binding potential. However, when these imperfections are much larger or much smaller than the bacterial size, the binding is not so strong [25].

Bacteria are incredibly diverse and the characteristics of the adhering bacterium itself are defined by its genome. Genetic variation that affects the surface composition of bacteria can occur within a species (i.e., differences between strains) as well as between species and may affect adhesion. Bacteria with hydrophobic surface would likely adhere better on hydrophobic surfaces while bacteria with hydrophilic properties adhere better on surfaces with charge [20].

### 3.2. Effect of Humidity on Antimicrobial Performance

The conditions imposed in the assay of antimicrobial performance set out in the JIS Z 2801 method differ from those reported under real conditions in hospitals [26]. Although such in vitro tests may not accurately recreate the desired conditions, they can be easily implemented and are useful in initial screening of new materials. As the standards do not accurately emulate the conditions experienced by bacteria during horizontal transfer in healthcare environments, researchers have sought to develop second-tier tests that perform antibacterial surface analysis under authentic conditions. Several studies report the bactericidal effect of copper in real hospital environments [27,28,29].

The deposition of bacteria on surfaces in hospitals can occur via an aerosol route after, for instance, coughing, sneezing and splashing. These could be simulated in a laboratory setting by generating bacterial aerosols. Despite the importance of this issue, only a few studies address it [26,30]. Ojeil et al. [26] deposited an *S. aureus* suspension on copper surfaces and measured antibacterial activity at three different conditions of temperature and relative humidity (RH): those stated in ISO 22196 (37 °C, 100% RH) and two authentic conditions at 20 °C with 40% or 50% RH and 100%. The authentic conditions generated an antibacterial activity around half of that measured under the standard conditions. Ojeil et al. [26] reported that the aerosols used to inoculate test materials in their study dried between 30 and 60 min, and the manuscript emphasises the importance of water (and hence the relative humidity) in enhancing the antimicrobial activity of copper. The higher antimicrobial activity reported for wet surfaces may lead to an overestimation of the bactericidal properties of the surface. For this reason, numerous studies have been focused on developing new tests at similar conditions as those detected in real conditions in hospitals [26,27,28,29,30,31].

**Figure 2:**
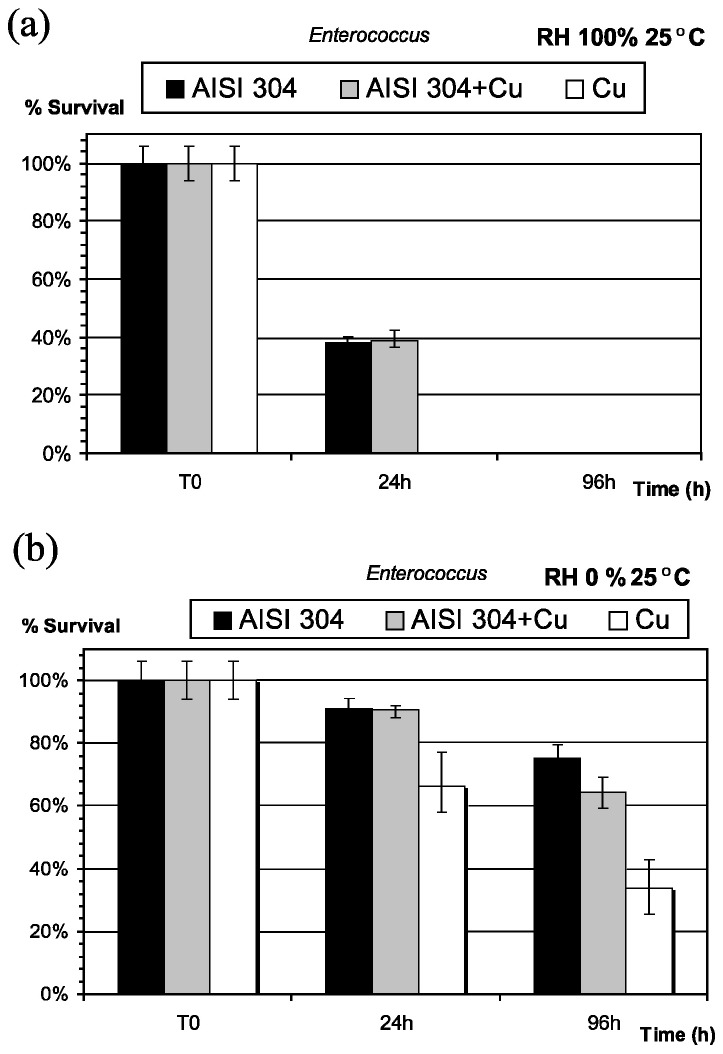
Survival of *E. faecalis* deposited as a bacterial aerosol on stainless steel AISI 304, stainless steel AISI 211 + 3Cu and copper) at 25 °C and (**a**) relative humidity of 100% and (**b**) relative humidity of 0% [30].

Robine et al. [30] reported similar outcomes when assessing the bactericidal properties of stainless steel grades and massive copper. An *Enterococcus faecalis* suspension was deposited on various materials: on stainless steel, stainless steel containing copper and on pure copper surfaces. They were subsequently incubated at 25 °C at a range of relative humidities. At 100% RH, bacteria were completely inactivated after 24 h but they survived at 0% RH for at least 96 h (Figure 2). Similarly, at the standardised high humidity and temperature conditions, a silver ion-containing material exhibited antimicrobial activity against *S. aureus* but no response was detected in standardised indoor environments (~24% RH and 20 °C) [32]. The lower antimicrobial activity values achieved at low humidities in these studies may suggest an overestimation of bactericidal properties of touch surfaces for evaluation in indoor conditions, such as hospitals, when studied using the standard methodologies.

Additionally, in order to replicate real conditions, not only the temperature and humidity are important but also the duration of the copper-microbe contact. For this reason, some authors have monitored experiments for long periods of time such as 10 weeks [27], 24 weeks [28] or more [29]. The major drawback is that these experiments are especially expensive in time and resources and therefore their widespread adoption is impractical.

The method for depositing cells on touch surfaces (i.e., wet plating and dry plating) also has an effect on the antimicrobial activity. For example, when *E. coli* cells are plated on copper by dry methods (dry plating), the accumulation of ions in the cells is larger than the concentration of copper ions in the cells when they are applied to copper coupons in wet conditions (i.e., wet plating) [10,33]. However, dry plating may not always be feasible as observed by Mathews et al. [34] since the spreading of cells with a cotton swab may destroy the cell’s structure and therefore a wet-plating technique would be preferred [4,35].

Although touch surfaces are of special relevance for the healthcare system due to their direct contact with human skin, other potential reservoirs of microorganisms are water pipeline systems. Cervantes et al. [31] tried to assess the bactericidal effect of three common plumbing materials (PVC, copper and Pyrex control) in aqueous solution. Several pathogen suspensions (including *S. aureus*, *P. aeruginosa*, *E. faecalis*, and *E. coli*) were studied at high and low inoculum densities in pipes of around 3.5 cm diameter, thus a very small proportion of the bacteria are in direct contact with the pipe surfaces. The experimental results showed high antibacterial activity with a reduction from 10^5^ CFU/mL to 0 CFU/mL within the first 2 h of contact for the copper samples while for PVC at 48 h 86% of the bacteria were recovered. These results clearly show that making drinking-water pipes out of copper is an effective way of enhancing water quality for human use.

### 3.3. Effect of Geometry, Chemistry and Physical Properties

#### 3.3.1. Bulk Samples

There are very few reports dealing with the antimicrobial behaviour of bulk metallic samples and most of them, as far as the authors are concerned, are focused on materials obtained by rapid solidification, i.e., bulk metallic glasses (BMGs). BMGs are suitable for touch surfaces in healthcare facilities due to their hardness and wear resistance (i.e., resistance against scratches), corrosion resistance attributed to the lack of grain boundaries and smooth surface finish that provides an aesthetic appearance. Only recently have BMGs been studied as potential materials for antimicrobial applications. In 2012, Lin et al. [36] studied the antimicrobial effect of Fe-based, Ni-based and Cu-based BMGs via a moist contact assay using *E. coli* bacteria. They found that the antibacterial effect seen after exposure to the three materials was practically the same, concluding that the most significant factor in the antimicrobial behaviour of the BMGs was not their composition but their non-crystalline structure. The antimicrobial effects in each of their assays was transient and was overwhelmed by denser inocula. This may in part be due to the assay measuring the effect of the glass on a culture to which it had partial and inconsistent contact rather than a culture applied directly to it.

**Figure 3:**
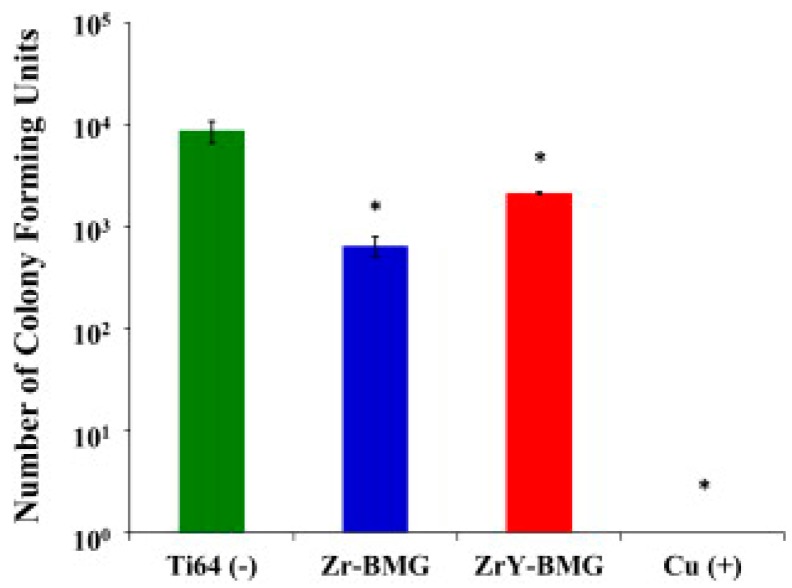
Growth of *E. coli* on exposure to Ti-6Al-4V, Zr_55_Al_10_Ni_5_Cu_30_ BMG, (Zr_55_Al_10_Ni_5_Cu_30_ BMG)_99_Y_1_ and pure copper after 4 h [11].

The antimicrobial behaviour of various Cu-containing Zr-based BMGs against *S. aureus* was recently investigated by Huang et al. [11]. Cu was alloyed with Zr to produce materials with sufficient good glass-forming ability to produce them in bulk shape [37]. These authors studied the bacterial killing of Zr_55_Al_10_Ni_5_Cu_30_ and (Zr_55_Al_10_Ni_5_Cu_30_)_99_Y_1_ (at. %) BMGs after 4 h of moist contact and compared the bacterial recovery with that observed on Ti-6Al-4V alloy and on pure Cu (Figure 3).

The highest killing activity was observed for Cu (no viable cells were observed after 4 h contact) followed by the Zr-BMG and finally the ZrY-BMG. These results were attributed to the higher concentration of Cu ions released from pure Cu than from its alloys. Dynamic immersion tests were also performed to model the application environment [11] for 1 day and they observed the formation of a *S. aureus* biofilm, suggesting that the bacteria can adhere to the BMGs. These results are contrary to the observation of Lin et al. [36] who claimed that bacterial (*E. coli* in their case) growth was prevented on metallic glasses due to their lack of adherence to the substrate.

#### 3.3.2. Coatings

Research with bulk samples is useful as a screening method to assess the antimicrobial performance of novel compositions since it is a fast technique that requires limited materials to produce samples. However, once the most promising compositions are selected based on the antimicrobial cost-effectiveness, they have to be produced as coatings in order to implement them in the healthcare sector. Thus to date, most of the research has been performed on coatings.

Over the years many researchers have reported that copper and copper alloys can effectively be used as antimicrobial touch surfaces [5,38,39]. Pure copper can eliminate 99.9% of bacteria after one hour, while for 60% copper, two hours were required to achieve similar values of antibacterial activity. These results highlight the interest in developing alloys with high Cu content in order to eliminate bacteria fast enough for the application sought. However, the number of reported Cu-rich thin film alloys is scarce and most of the research is basically focused on the Cu_90_Ti_10_ [40,41,42] alloy.

**Figure 4:**
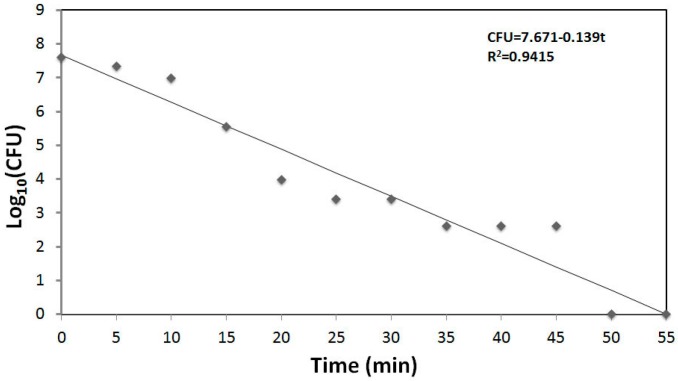
Kinetics of bacterial killing on thin copper films. A suspension of *Staphylococcus aureus* was deposited on a thin copper film supported by a stainless steel support under ambient conditions (22 °C). Samples were taken periodically and survivors were quantified by culturing serial decimal dilutions on LB agar plates (unpublished results from the authors).

The antimicrobial performance not only depends on the alloying elements added to copper but also on the coating techniques and therefore on the microstructure. Different spray techniques have been reported to be feasible to fabricate coatings: plasma sprays, wire arc sprays and cold sprays. An economic consideration is the production of a durable coating with a long working life and using the minimum of materials, thus the production of thin films has received much attention. The process of choice for deposition of metallic thin coatings in a wide range of industrial applications is magnetron sputtering. We have recently considered the antimicrobial performance of pure copper thin film produced by magnetron sputtering against the pathogen *S. aureus* (unpublished results). Much like the methodology described in ISO 22196:2011 and JIS Z 2801:2010, a suspension of *S. aureus* was placed on the copper surface, and samples of the suspension were taken at intervals of 5 min for an hour. A denser inoculum than the one described in the standard (by two orders of magnitude) was used and the procedure was carried out under ambient laboratory conditions. Bacteria were recovered from the suspension in contact with the film and were quantified by analysing serial decimal dilutions cultured on agar plates (Figure 4). The number of bacteria recovered can be given as the number of Colony Forming Units (CFU). The CFU recovered decreased exponentially from 4.6 × 10^7^ to 0 when the contact time was increased from 0 to 50 min. The profile is complicated by the fact that the *S. aureus* colony forming unit is rarely a single cell but these grow as a clump in liquid culture. Each clump, which may contain 10 or more cells, might form a colony if only one of the clumped cells survives; the kinetics of death therefore appear complex.

An effective technique for producing amorphous alloys is to make them as thin films by magnetron sputtering [43,44]. Amorphous alloys are important since they can be used as precursor materials for controlling the type and volume fraction of crystalline phases, which enables tuning of the mechanical and antimicrobial properties. Over the years, the antimicrobial activity of various thin film alloy compositions has been studied. Most of the papers are focused on Zr-based or Ti-based alloys such as Zr_53_Cu_30_Ni_9_Al_8_ [17], Zr_43_Cu_40_Al_9_Ag_8_ [45] and Ti_40_Cu_30_Pd_14_Zr_10_ [46], for which complete elimination of the cultivated bacteria only occurs after 24 h. Differences in antimicrobial activity were mainly attributed to variations in copper content, i.e., the antibacterial activity increases with increasing copper content [47], since a higher concentration of copper should release more Cu ions (i.e., dissolution of Cu).

With regard to evaluation of differences in antimicrobial activity of the materials, various methodologies have been reported to date. Some authors such as Chu et al. [17] used the plate count method where the number of bacterial colonies that grew on each agar plate is counted and compared to the control. Other methods are viz. agar diffusion method, solution suspension and wet interfacial contact method [45,46]. The agar diffusion plate test generates a zone of inhibition on a confluent bacteria lawn, indicating the diffusion of Cu ions from the sample to a threshold inhibitory concentration, thus the diameter of the zone indicates efficiency in release of the ions. The wet interfacial method involves direct contact between the coating and the viable bacteria can be visualised by Epi-fluorescence microscopy.

There are also differences in the conditions at which antimicrobial tests have been carried out, which makes it difficult to compare the results of the different authors. While some studies were carried up to 24 h [42,46], other studies have been done for various longer periods (ranging from 3 to 20 days) [17,40,45].

Other antimicrobial elements such as Ag or Zn are able to eliminate pathogens in thin films. For example, Chu et al. [48] observed that the antimicrobial performance of Ag-containing ZrTiAl and AlAgTi thin films is similar to that of a pure Ag coating. In several other studies Ag was incorporated as an element in thin films [49,50,51] to tune the antimicrobial performance, but the alteration of another parameter (roughness, size of crystal formation, etc.) makes it difficult to assess whether the killing effectiveness can be only attributed to compositional differences. Sometimes it can be useful to make composites of various materials to enhance the flexibility to tune the behaviour (Ag-TiO_2_ composites [52,53,54,55] or Ag nanoparticles [56], etc.) but a detailed description is beyond the scope of this manuscript.

#### 3.3.3. Physical Properties of Metallic Coatings

The studies previously described have been mostly focused on optimising the chemical composition of materials to control their antimicrobial behaviour. Nevertheless, the physical properties of thin film surfaces also have an effect on pathogen elimination rate. A useful parameter to predict the antibacterial activity is the wettability [42], which depends on the surface roughness, surface-free energy or surface structure. Consequently, understanding the role of these parameters in the antibacterial properties of thin films is an issue of utmost importance.

In terms of wettability, contact angle measurement is the preferred method to measure surface-free energy. High contact angles (i.e., the angle between the surface of a liquid and the outline of the liquid-solid contact surface) are attributed to hydrophobic behaviour, while materials showing lower values present better adhesion properties to the material [42]. Hydrophobic materials (contact angles >65°) are usually recommended for antimicrobial applications since such surfaces inhibit cell attachment [42].

Surface roughness can be controlled by careful selection of the sputtering conditions in the magnetron sputterer, which is a useful physical vapour deposition method to produce thin films. Chen et al. studied the effect of coating surface roughness obtained at different sputtering power conditions on antimicrobial activity [45] and observed that surfaces generated under lower power conditions exhibited a higher antibacterial capability compared to those generated with higher power. Nevertheless, this study did not investigate the relationship between sputtering power and surface morphology in depth. For this reason, a complete understanding of the reasons which lead to better antibacterial properties was not achieved. On the other hand, Stranak et al. investigated the role of several sputtering techniques on the bactericidal characteristics of Cu_90_Ti_10_ alloy [41]. Three different magnetron sputtering techniques were analysed (direct current magnetron sputtering: DC, dual magnetron-sputtering: Dual-MS and dual high power impulse magnetron-sputtering: Dual-HiPlM) and their antibacterial properties were compared. Dual-HiPlM was regarded as the most promising technique for antibacterial applications because the thin film produced thus released the highest concentration of copper ions (i.e., 40% more than in DC and Dual-MS) [41].

Among the metallic coating fabrication techniques, magnetron sputtering is the most broadly used, as indicated by the large number of manuscripts found in the literature. Even for the same sputtering conditions, a small change in composition can result in differences in the nucleation process and surface topography. For example, Wojcieszak et al. [51] reported that magnetron-sputtered Ti-Ag and Nb-Ag coatings exhibited extremely different nucleation of the crystalline phases, which resulted in a surface roughness of 2 nm for Ti-Ag and 22 nm for Nb-Ag. Larger surface roughness can be attained using wire arc spray [18]. The roughness of the copper coatings was studied and correlated with the antibacterial properties. In this technique, the coating is fabricated by propelling molten droplets at high velocities to the substrate surface in a high oxygen atmosphere. The roughness achieved in this technique may be five orders of magnitude higher (from 1 nm to 100 μm) than that obtained using the conventional magnetron sputtering technique. The antimicrobial activity of these coatings was about 92%, compared with the 99.999% obtained using magnetron sputtering [48]. It is important to note that wire arc coating is done in a high oxygen atmosphere [18] and therefore results in the formation of surface oxides. When significant surface oxidation takes place, micron level roughness might be covered by the oxide layer and the roughness may not have an influence on the antimicrobial performance. The formation of oxides is therefore important and will be covered in the next section.

### 3.4. Oxides

Metal oxides are important for their unique physicochemical properties and antibacterial activity. Among all oxides, those detected on copper, silver and zinc have special relevance since they are antimicrobial materials. There is an increasing interest in these elements and especially in copper due to its relatively low cost and antimicrobial efficiency.

**Figure 5:**
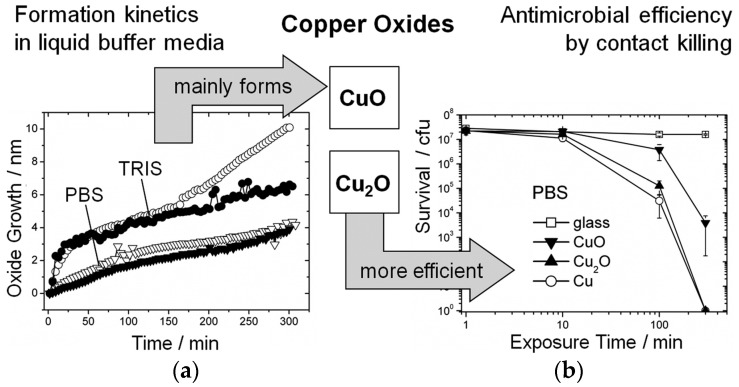
(**a**) Plot showing oxide growth versus oxidation time in PBS and in Tris; (**b**) Plot survival of *E. hirae* exposed to glass, Cu, CuO and Cu_2_O at different times [57].

The antimicrobial mechanism of copper oxides [57] is not understood in detail but seems to be the result of several co-acting mechanisms where the primary cause is direct contact between bacteria and metallic copper since copper ions can induce severe damage to the bacterial envelope. According to Hans et al. [58] the formation of copper oxides that are soluble in aqueous phase followed by release of copper ions from the oxides are key factors that make copper antimicrobial. In order to assess the contact killing activity of Cu oxides, Hans et al. [57] grew oxide layers in phosphate buffered saline (PBS) or Tris-HCl (pH 7) for 300 min (Figure 5a) and measured the number of survival of bacteria (Figure 5b) versus exposure time in glass, Cu, Cu_2_O and CuO. The antibacterial efficacy for pure Cu and Cu_2_O were very similar but slightly smaller for Cu_2_O; the activity for CuO was smallest. The buffer medium Tris-HCl was observed to induce faster copper ion release than PBS and therefore higher killing efficiency.

The antimicrobial behaviour of other metal oxides is also of interest but studies are very scarce. Most consider oxidation on particles/nanoparticles and therefore it is difficult to assess whether the antimicrobial performance is attributed to the oxide or to the particle size. However, Rebelo et al. [50] developed Ag_x_O thin films and compared the antibacterial effect with that of pure Ag thin films to establish whether the killing efficiency can be improved by promoting the formation of oxides. Thus, antibacterial activity of the materials was investigated through halo inhibition zone test using *Staphylococcus epidermidis* and *S. aureus*. For both bacteria, zones of inhibition were observed with Ag_x_O samples but not with pure Ag. The format of the assay suggests that the release of Ag ions is enhanced in the AgxO samples as the significant bacterial killing occurs at a distance to the sample. However, the authors speculate that not only the presence of silver oxide phases but also their heterogeneous growth forming ‘islands’ could promote the antimicrobial activity due to enhancing interactions between the thin film surface and bacteria.

As in the case of Cu metal, the antimicrobial activity of Ag depends on the presence of ions. In order to promote Ag ionisation, the oxidising compound sodium hypochlorite can be used in Ag-containing coatings. This process is called silver activation and has been successfully used to promote the antimicrobial effect of the coatings [49]. Another method for eliminating pathogens, photocatalysis disinfection using ultraviolet light, involves promoting the release of reactive oxygen species resulting from redox reactions at the surface of the TiO_2_ films [59]; this also has self-cleaning properties [59,60,61]. The antimicrobial properties of these films can be increased with the addition of Ag and Cu [52,53,54,55].

**Figure 6:**
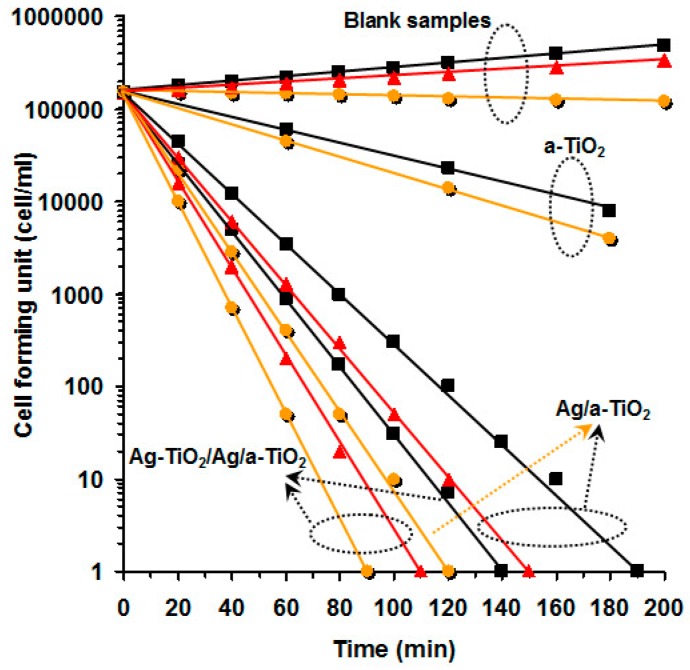
Killing of *E. coli* exposed to a-TiO_2_, Ag/a-TiO_2_ and Ag-TiO_2_/Ag/a-TiO_2_ photocatalyst thin films as compared to blank (control) sample, in dark (■) or irradiated with visible light (▲) or solar light (●) [52].

Page et al. [54] were one of the first researchers to develop an Ag-TiO_2_ composite for antimicrobial purposes. The Ag-doped TiO_2_ films exhibited more photocatalytic and antimicrobial activity than TiO_2_ coatings and did not show activity against bacteria in the dark, suggesting that their performance was not due to the passive release of silver ions. It was suggested that these composite films might be useful as coatings on hard surfaces in hospitals. Outstanding results were also obtained by other authors when Ag particles were added. For example, 1 wt % Ag addition to TiO_2_ was reported to reduce the time to destroy *E. coli* irradiated with ultraviolet A from 65 to 16 min [62]. Akhavan [52] went one step further and developed a three-layered thin film (Ag-TiO_2_/Ag/a-TiO_2_). The author studied the survival of *E. coli* in a medium containing a-TiO_2_, Ag/a-TiO_2_ and Ag-TiO_2_/Ag/a-TiO_2_ photocatalyst thin films in dark, visible and solar light irradiations (Figure 6). In each case, the population of viable bacteria decreased exponentially but the rate depended on the thin film composition and the illumination used. For a-TiO_2_ the rate of reduction of viable bacteria was up to 20% in solar light irradiation. A similar trend was observed in Ag/a-TiO_2_ and Ag-TiO_2_/Ag/a-TiO_2_ when exposed to solar light instead of darkness. For visible light the rate of reduction was intermediate between darkness and under solar light.

## 4. Operational Challenges, Cleaning and Disinfection

The cleaning regimes used in hospitals and how they might influence the antimicrobial activity of touch surfaces requires careful consideration. Build-up of organic deposits on these surfaces during use are not only to be avoided for simple aesthetics but they would provide a protected and likely more efficient substrate for indirect attachment of bacteria to the surface. Despite the relevance of the influence of disinfectants and detergents on antimicrobial performance, little attention has been paid to this topic [63]. This is especially important considering that HAI pathogens such as *S. aureus*, *Clostridium difficile* or *Acinetobacter baumannii* can survive for weeks in hospital environments [63,64]. As a result, doubts regarding current cleaning procedures have arisen and led to the definition of the cleaning standards and conditions for a disinfectant to be “ideal” [64].

Cleaning thoroughness in patients’ rooms and of frequently touched surfaces have usually been assessed by visual inspection [65,66,67,68,69]. In contrast to visual inspection, when microbiological sampling [68], bioluminescence [67,68,69] and ultraviolet marking have been employed to understand the disinfection thoroughness in High Touch Objects, most of the surfaces which seemed “clean” by visual inspection did not meet benchmark values. Malik et al. [68] revealed that 90% of the tested surfaces did not meet the required benchmark, while Griffith et al. [67] showed that 70% to 76% of the investigated sites were “unacceptable” after cleaning. Consequently, environmental hospital cleaning inspection may be subjected to changes in future.

Regular and effective cleaning of antimicrobial touch surfaces promotes direct contact between the pathogen and the metal surface and therefore optimises antimicrobial effectiveness. However, chemicals from the cleaning products may change the surface chemistry. This is exemplified in the work undertaken by Airey et al. who researched the influence of continual cleaning of copper and steel surfaces [70]. In this study, a 1% sodium hypochlorite and 70% industrial methylated spirit were used. During the five days of the testing, steel and copper coupons were subjected to daily cycles of cleaning. After the trial period, a build-up of cells and soil was reported on the copper surfaces. In light of these results, it is clear that proper cleaning standards have to be developed for each material. Due to the global interest in the issue, there are different organisations such as the Copper Development Association [71], a n not-for-profit trade association that supports and promotes copper usage in the UK and the US, developing guidance on cleaning and disinfection of antimicrobial copper alloy touch surfaces.

When analysing the effect of disinfectants in the cleaning effectiveness and the hazards derived from their use, one has to take into consideration that disinfectants are complex solutions of multiple components (see Table 2). The most abundant component is water, from 15% to 95%. The content of acid can be very high, from 0.1% to 35%, and therefore might be taken into consideration when cleaning antimicrobial surfaces due to its potential reaction with copper. For example, commercial hydrochloric acid and sulphuric acid can have a destructive effect on copper surfaces during the cleaning process [72], which can be suppressed with the addition of corrosion inhibitors to the formulation. The concentration of the sodium hypochlorite disinfectant, (also known as bleach when dissolved in water) ranges from 0.1 to 10 wt %. Despite the increasing availability of other disinfectants (see Section 3.1), hypochlorites are widely used in hospitals due to their broad and rapid antimicrobial activity and low cost [73]. When selecting disinfectants one also has to consider their potential negative impact on the environment. For example, benzalkonium chloride is a quaternary ammonium compound broadly used as a disinfection compound in industries and households and, as a result, is present in waste. These residues exhibit high toxicity for aquatic and terrestrial fauna and therefore its use will probably be limited in the next decades [74]. Considering that disinfectants are the active substance in cleaning products, they will be the focus of the following sections. Other parameters such as the presence of water and sweat, mostly from hand contact, and the pH of the cleaning products can potentially have an effect on the performance of antimicrobial materials (basically copper, zinc and silver) and therefore will be also discussed.

### 4.1. Active Substances

The number of disinfectants used to destroy bacteria and other microorganisms can be classified into four main groups [75,76]:

(a) Hydrogen peroxide solutions
(b) Chlorine-releasing compounds (hypochlorite)
(c) Alcohols (ethanol) and aldehydes (formaldehyde)
(d) Quaternary ammonium compounds (benzalkonium chloride)

The efficiency of these disinfectants are dependent upon two different conditions: (1) direct contact of liquid disinfectants with the bacteria deposited on surfaces upon cleaning; and (2) once the disinfectant has dried up, interaction between the chemically modified touch surface (i.e., oxide) and bacteria. Because of these links, not only is it important to know the antimicrobial efficiency of the material itself but also how this effectiveness may be disrupted by surface modification of the material due to the chemicals contained in cleaning products.

#### 4.1.1. Hydrogen Peroxide

*Hydrogen peroxide (H_2_O_2_)* is widely used for antimicrobial disinfection because it is effective against many microorganisms, including *E. coli* [77,78], *S. aureus* [77,78,79,80], *Bacillus atrophaeus* [81], *A. baumannii* [82], *C. difficile* [82] and oral streptococci [83] and does not generate significant environmental and toxicity effects [76,84]. The chemical can also be used as an aerosol or vapour in automatic systems [81,82]. Dry mist-generated hydrogen peroxide disinfection systems are able to release controlled amounts of a H_2_O_2_ solution, disinfecting complete rooms automatically. These strong antibacterial activities, simple mode of use and quick application makes hydrogen peroxide an effective and attractive chemical for hospital disinfection. However, despite all these advantages, the information in the literature describing how hydrogen peroxide and antimicrobial materials interact is very scarce. In the case of copper, DeNardis et al. had reported the formation of several copper oxides at surfaces due to the application of hydrogen peroxide [85]. The nature of these copper species is dependent on the contact time between surface and disinfectant. They observed the formation of a Cu_2_O/Cu layer during the first 5 min of exposure to H_2_O_2_ (at 1 wt %). Nevertheless, new copper species were formed (Cu(OH)_2_, CuO and Cu_2_O) as the exposure continued. As has been shown before (Section 3.3 Oxides, Figure 4), the antimicrobial activity of copper surfaces tends to decrease as the ratio of oxygen to copper of the oxides formed on the surface increases [57]. For Cu_2_O, the antimicrobial performance is similar to that of Cu while for CuO it is not much lower for exposure times up to 100 min. However, for longer exposure times, the difference in antimicrobial performance increases and it is therefore of utmost importance to know the nature of the oxides formed.

Hydrogen peroxide also has an effect on the surface of the other commonly used antimicrobial materials, silver and zinc. As related in Section 3.3, the antimicrobial behaviour of silver is attributed to the release of toxic ions from the oxide, not from the silver itself [86]. This explains the growing interest in developing silver nanoparticles to enhance antimicrobial performance [87]. Although silver is advantageous from the point of view of its resistance to corrosion (the antimicrobial activity decreases with the formation of oxides) The observation that silver is antimicrobial may therefore have been misinterpreted [87]. The high cost of silver is limiting the implementation in the healthcare sector, which may explain the scarce literature available about the interaction between silver and common chemicals. In regards to the influence of hydrogen peroxide, an aqueous solution of silver ions and hydrogen peroxide was reported to reduce biofilm formation [88]. Armon et al. [88] discovered that a solution of H_2_O_2_ was only able to prevent the formation of biofilm in a pipeline for up to 50 h, though addition of silver ions stabilised the short-term biofilm elimination provided by the disinfectant indicating a degree of synergy between both materials. Although the studies were developed for the use of the solutions in drinking and wastewater pipes, they encourage more in-depth research of this type of solution for touch surface disinfection. Additionally, zinc oxidises and the ions released also exhibit antimicrobial properties [89]. Moreover, when light interacts with zinc oxide, a synergistic phenomenon takes place and the antimicrobial activity is enhanced [90,91]. However, the bactericidal effect of this oxide may not be completely caused by the presence of zinc ions but also due to the generation of hydrogen peroxide. According to some researchers, ZnO suspensions are able to produce hydrogen peroxide [92,93]. Clearly, the effect of this chemical in the antimicrobial activity of ZnO needs more work.

#### 4.1.2. Chlorine-Releasing Compounds

Chlorine-releasing compounds are characterised by their broad antimicrobial spectrum, rapid action, high solubility, persistence in potable water, relative non-toxicity and lack of dangerous residuals, deodorizer activity, lack of colour and staining, non-flammability and low cost [73]. Most of these properties are necessary or highly recommended in disinfectants. As a result, chlorine and chlorine-based disinfectants such as sodium hypochlorite (i.e., NaClO) are widely used in disinfection. The major drawback of both chlorine and hypochlorite is that they are highly corrosive and oxidising chemicals [94]. They can corrode a wide range of materials including Cu, Zn and Ag and alter their antimicrobial effectiveness. Due to the importance of the NaClO in copper corrosion, two different tests were carried out by Montes et al. [95]: a static test involving the immersion of copper into a hypochlorite solution in tap water and a dynamic test in which a continuous flow of the same solution was used. In the static test, after 45 days’ immersion, the main copper species that developed was Cu_39_S_28_, but also some Cu_2_O was formed [95]. It has to be noted that there is no apparent source of sulphur in the experiments. However, tap water was used in the experiments which may be the source of the sulphur species. Nevertheless, the water source was not investigated. After 8 weeks of interaction, different species were reported depending on the testing temperature: malachite (Cu_2_CO_3_(OH)_2_) at 50 °C and tenorite (CuO) at 70 °C [95]. All these copper compounds exhibit antibacterial behaviour to a greater or lesser extent, including copper sulfate [96].

Hypochlorite (NaClO), a chlorine-containing chemical, reacts with Zinc (Zn) to form Zinc Oxide (ZnO) and NaCl (salt) but does not result in silver oxide formation (2Ag + 2NaOCl = 2AgCl + 2Na + O_2_). Most of the manuscripts in the literature deal with ZnO and silver [90,91,97] as particles or nanoparticles. Their antimicrobial behaviour depends on colloid particle size and therefore the antimicrobial effectiveness due to oxidation is masked by this parameter. However, it is interesting that for similar particle size, higher antimicrobial activity against *Streptococcus mutans* is attained with silver nanoparticles at lower concentrations than for gold or zinc oxide [98], which suggests that the composition has more influence than the colloidal particle size.

Chlorine is commonly used for disinfection; for this reason it is useful to know the influence of these chemicals when in contact with the pathogens normally found in hospitals [99]. Probably due to the overuse of this chemical for cleaning purposes, pathogen resistance to high chlorine concentrations has been reported [100]. The chlorine antibacterial efficiency can be increased by incorporating copper and silver ions as reported by Kim et al. [101] and Landeen et al. [102].

#### 4.1.3. Alcohols and Aldehydes

In chemistry, an alcohol (e.g., ethanol, isopropanol etc.) is a chemical compound in which a saturated carbon atom is bound to a hydroxyl group (−OH). On the other hand, aldehydes (i.e., glutaraldehyde, formaldehyde) are organic compounds containing a formyl group (−COH), commonly formed due to the oxidation of alcohols. Exceptional works can be found in the literature about the bactericidal efficacy of alcohols and aldehydes [103]; for this reason, the main focus of this section will be the role of these disinfectants in modern hospital sterilisation.

The use of alcohols and aldehydes has been focused on improving hand disinfection [103]. Until this point, all the methods of increasing the antimicrobial activity of touch surfaces have been focused on connection to changes in morphology or composition. Hand disinfection may not have a direct influence on increasing the bactericidal effect of these surfaces. Nevertheless, control of skin pathogens may complement the effects displayed by antimicrobial materials. In this regard, commercial hand disinfectants such as Sterillium Gel^®^ have displayed interesting antibacterial properties [104]. The increase in number of pathogens resistant to common biocides is encouraging the development of new hand disinfectant gels. In order to improve the efficacy of ethanol biocides, silver [105] and zinc [106,107] have been added. Experimental rubs with these chemicals revealed log_10_ survival rates greater than 3, which comply with the standards required from the Food and Drug Administration imposed in the United States.

Due to the frequent use of alcohol-based hand rubs and disinfectants by healthcare workers, these may pose a risk of skin irritation [108]. Discomfort caused by skin irritation may compromise hand disinfection compliance in healthcare workers. Subsequently, an increase in pathogen survival may be expected. Healthcare disinfectant manufacturers are aware of this issue, and, as a result, non-alcohol disinfectant rubs are being produced. As an example, a commercial copper-based gel (*Xgel*) has been developed as a possible alternative [108]. In a recent study, *Xgel* revealed high bactericidal properties when compared to other commercialised hand rubs.

#### 4.1.4. Quaternary Ammonium Compounds

These compounds can be used in multiple applications including disinfectants, surfactants and softeners [109]. From the point of view of disinfection, they are bactericidal for a wide range of pathogens such as *E. coli* [110,111], *S. aureus* [112,113,114], S. *mutans*, *B. subtilis*, *P. aeruginosa* [114] and the fungal pathogen *C. albicans* [112,114].

The interaction between metal ions and quaternary ammonium compounds has been studied by Harrison et al. [115], who analysed the interaction between different solutions of metal ions (Ag^+^, Cu^2+^, Al^3+^, SeO_3_^2−^, Zn^2+^) and commercial disinfectants (Polycide, Stabron 909, isopropanol and Virox). They found a synergistic effect of copper ions (Cu^2+^) and quaternary ammonium compounds such as benzalkonium chloride, cetylpyridinium chloride, cetalkonium chloride or myristalkonium chloride. The addition of copper ions to these chemicals synergistically raised the antibacterial activity against *P. aeruginosa* biofilms up to over 100-fold.

Quaternary ammonium salts can influence the surface properties of copper. Hegazy et al. [116] revealed that two quaternary ammonium compounds showed corrosion inhibition of copper in a 1 M nitric acid solution (HNO_3_) by ten-fold. Corrosion inhibition was reduced when the temperature increases from 25 °C to 55 °C [116]. From the point of view of quantitative research, the publication from Hegazy et al. [116] is one of the most interesting in copper corrosion. The effect of temperature and inhibitor quantity in copper corrosion has been accurately calculated although a more detailed chemical analysis of the developed species during the corrosion stages of the substrate would have been useful.

### 4.2. Water and Sweat

Touch surfaces are continuously subjected to fingerprint residues and sweat. For this reason, there is interest in understanding the corrosion resistance of antimicrobial touch materials to hand contact. In the literature, early consideration of these issues can be found [117] and the topic has been also more recently covered by Bond [118]. The corrosion resistance of several metallic surfaces and alloys (Aluminium, Brass, Copper, Gold, Magnesium, Silver, Steel, Zinc, etc.) to fingerprint residue was compared [118]. Although the species formed during hand contact were not analysed, it illustrates the differences in corrosion resistance among several metals, including three antimicrobial materials that have been extensively discussed in the previous section: copper, silver and zinc. Under the same conditions, fingerprints were more defined in copper, meanwhile silver and zinc surfaces did not show a detailed fingerprint development [118]. In light of these results, copper seems to be the most prone to corrosion mediated by skin residues among the bactericidal elements discussed.

A remarkable study about colour stability during prolonged hand contact and corrosion resistance in synthetic sweat was conducted by Fredj et al. [119]. In this research, baton tests were performed on pure copper (C110), brass (C260), bronze (C510), a cupronickel alloy (C706) and a copper alloy of nickel silver (C752). A layer of Cu_2_O was found on all the materials analysed; however, the thickness of this oxide was highly dependent on the composition (i.e., 50 nm for the nickel silver alloy and 230 nm for pure copper) [119]. Interestingly, corrosion test in synthetic sweat revealed that the most prone to corrosion were the cupronickel alloys and the nickel silver alloys, which maintained the lustrous appearance after two years of hand contact. From these results it can be concluded that high nickel content alloys would be preferred due to their aesthetics. Nevertheless, nickel is one of the most common metal allergens [120], which should clearly be taken into consideration when selecting elements for touch surfaces. Selection of the proper disinfectants is also important when cleaning Cu-rich touch surfaces since the formation of CuO upon cleaning would dramatically decrease the bactericidal effect of the touch surface.

### 4.3. The Influence of pH on Corrosion

Another relevant factor that plays a major role in metal corrosion is the acidity or alkalinity of the applied solution (i.e., pH) [121,122,123]. Copper is highly resistant to most alkaline and acid substances. The main reason behind copper’s resistance to such corrosion is the formation of a protective layer of Cu_2_O during the early stages of chemical interaction [123]. Daniels et al. [124] investigated the changes in roughness of several copper coupons submerged in an aqueous solution at pH 6.5, 7, 8 and 9. After 24 h, AFM imaging of these coupons revealed an increase in roughness caused by the formation of a Cu_2_O layer (0.26 µm for pH 6.5, 0.5 µm for pH 7 and 0.12 for pH 8). However, copper is heavily corroded by the presence of ammonium-based chemicals [94].

Silver generally resists oxidation but, when it is in contact with organic or inorganic sulfuric and with chloride species, a surface tarnish film forms [122]. Similarly, in the absence of complexing ions, silver is immune to corrosion for almost all the pH range. For this reason, silver is stable in some acids such as hydrochloric acid, acetic acid or phosphoric acid [122]. However, when silver is in contact with highly corrosive oxidising acids (such as nitric acid), a chemical reaction takes place that leads to the formation of nitric oxide (NO), silver nitrate (AgNO_3_) and water.

Unlike copper and silver, zinc is soluble in acids with pH lower than 5 and in alkaline solutions with pH higher than 12.5 [121]. For diluted chemicals, the corrosion rate of zinc is dependent on the impurities of the material. Commercial zinc in contact with a diluted acid will start to corrode at a slow rate but the zinc sponge precipitated during the corrosion will increase the rate of this reaction [121]. On the other hand, zinc can be used for storage of milder chemicals such as detergents and organic chemicals [121].

## 5. Conclusions and Future Directions

Metal alloy touch surfaces are widely used in hospitals and the healthcare sector and therefore tuning their properties can contribute to hospital hygiene and infection control. However, the use of touch surfaces should be viewed as a useful and complementary component of a comprehensive hygiene regime rather than an alternative to other methods. The antimicrobial activity and cost-effectiveness of copper makes this metal of particular interest but the material properties of its alloys need to be more thoroughly understood in order to deliver the best compromise of durability, aesthetics and long-lasting antimicrobial activity. Our review has highlighted the need for the development of a standardised suite of assays that may rapidly refine promising alloy compositions that meet well-defined benchmarks in terms of antimicrobial activity and its retention in the face of the environmental and operational challenges we have discussed. The influence of the method of coating application and its effect on the physical surface properties is of utmost importance to deliver the best possible protection for patients in the modern world where therapeutic antibiotics is facing serious challenges from the rapidly evolving infection risk. Controlling the surface chemistry of touch surfaces combined with proper cleaning products and procedures has been proven as the best method to attain highest antimicrobial performance. The ability to tune surface patterning of acrylic touch surfaces has recently shown the potential to drive a major advancement in limiting microbial transmission [125]. A potential technique that may drive future improvements in antimicrobial performance is micropatterning metallic thin films; however, still many challenges would need to be overcome before they can be effectively implemented. Among them, it is important to prevent changes in surface chemistry, textures and structures over the time that may result in a dramatic drop of antimicrobial activity.

In this manuscript we have developed a comprehensive review of the most fundamental issues on antimicrobial behaviour of metallic touch surfaces in healthcare environments. From these results the following conclusions have been drawn:

- There is a need for appropriate standardised antimicrobial tests for touch surfaces.
- There is a lack of information about the effect of corrosion products on the antimicrobial behaviour of touch surfaces (there is either information about the effect of direct contact between cleaning products and microbes or the effect of cleaning products on the chemistry change of cleaned surfaces).
- Very few antimicrobial tests have been performed under real conditions with long-term exposure to recreate hospital and other healthcare environments. Differences in humidity and temperature conditions across the globe may result in different outcomes.

## Figures and Tables

**Table 1:** Summary of antimicrobial testing standards.

Standard	Name	Country of Application	Pathogens Analysed	Antibacterial Benchmark
JIS Z2801:2010	Antibacterial products—Test for antibacterial activity and efficacy.	Japan	*S. aureus*, *E. coli*	No
ISO 22196:2011	Measurement of antibacterial activity on plastics and other non-porous surfaces.	Europe	*S. aureus*, *E. coli*	No
US EPA ^1^	Protocol for the Evaluation of Bactericidal Activity of Hard, Non-Porous Copper Containing Surface Products	USA	*S. aureus*, *P. aeruginosa*	Yes ^2^

^1^ This document is released as part of a consultation process in the development of the standard. ^2^ Considered as a sanitizer when more than 99.9% of the pathogens are eliminated.

**Table 2:** Usual composition of cleaning products [75].

Component	Concentration (*w*/*w* %)	Examples of Compounds
Disinfectant (active substance)	0.1–10	Benzalkonium chloride, Sodium hypochlorite
Surfactant	0.1–10	Benzenesulphonic acid, dodecyl-, sodium salt
Base	0.1–25	Sodium hydroxide, Potassium triphosphate
Complexing agent	5.0–30	Pentasodium triphosphate, EDTA
Corrosion inhibitor	1.0–10	Disodium metasilicate
Solvent	0.1–10	2-Propanol
Perfume	0.002–1	Citrus oils, eucalyptus oil
Pigment	0.01–2	
Acid	0.1–35	Phosphoric acid, Citric acid
Diluent	15.0–95	Water

## References

[1] European Centre for Disease Prevention and Control (2013). Point Prevalence Survey of Healthcare Associated Infections and Antimicrobial Use in European Acute Care Hospitals.

[2] European Centre for Disease Prevention and Control (2015). Antimicrobial Resistance Surveillance in Europe 2014. Annual Report of the European Antimicrobial Resistance Surveillance Network (EARS-Net).

[3] Dollwet H.H.A., Sorenson J.R.J. (1985). Historic Uses of Copper-Compounds in Medicine. Trace Elem. Med..

[4] Wilks S.A., Michels H., Keevil C.W. (2005). The survival of *Escherichia coli O157* on a range of metal surfaces. Int. J. Food Microbiol..

[5] Santo C.E., Lam E.W., Elowsky C.G., Quaranta D., Domaille D.W., Chang C.J., Grass G. (2011). Bacterial Killing by Dry Metallic Copper Surfaces. Appl. Environ. Microbiol..

[6] Warnes S.L. (2014). Laboratory Studies to Investigate the Efficacy and Mechanism of Action of Copper Alloys to Kill a Range of Bacterial Pathogens and Inactive Norovirus. Ph.D. Thesis.

[7] Warnes S.L., Highmore C.J., Keevil C.W. (2012). Horizontal transfer of antibiotic resistance genes on abiotic touch surfaces: Implications for public health. mBio.

[8] Warnes S.L., Keevil C.W. (2013). Inactivation of norovirus on dry copper alloy surfaces. PLoS ONE.

[9] Borkow G., Gabbay J. (2009). Copper, An Ancient Remedy Returning to Fight Microbial, Fungal and Viral Infections. Curr. Chem. Biol..

[10] Grass G., Rensing C., Solioz M. (2011). Metallic Copper as an Antimicrobial Surface. Appl. Environ. Microbiol..

[11] Huang L., Fozo E.M., Zhang T., Liaw P.K., He W. (2014). Antimicrobial behavior of Cu-bearing Zr-based bulk metallic glasses. Mater. Sci. Eng. C Mater..

[12] Vincent M., Hartemann P., Engels-Deutsch M. (2016). Antimicrobial applications of copper. Int. J. Hyg. Environ. Health.

[13] Tong S.Y., Davis J.S., Eichenberger E., Holland T.L., Fowler V.G. (2015). *Staphylococcus aureus* infections: Epidemiology, pathophysiology, clinical manifestations, and management. Clin. Microbiol. Rev..

[14] Croxen M.A., Finlay B.B. (2010). Molecular mechanisms of *Escherichia coli* pathogenicity. Nat. Rev. Microbiol..

[15] Driscoll J.A., Brody S.L., Kollef M.H. (2007). The epidemiology, pathogenesis and treatment of *Pseudomonas aeruginosa* infections. Drugs.

[16] (2016). Protocol for the Evaluation of Bactericidal Activity of Hard, Non-porous Copper Containing Surface Products.

[17] Chu J.H., Lee J., Chang C.C., Chan Y.C., Liou M.L., Lee J.W., Jang J.S.C., Duh J.G. (2014). Antimicrobial characteristics in Cu-containing Zr-based thin film metallic glass. Surf. Coat. Technol..

[18] Sharifahmadian O., Salimijazi H.R., Fathi M.H., Mostaghimi J., Pershin L. (2013). Relationship between surface properties and antibacterial behavior of wire arc spray copper coatings. Surf. Coat. Technol..

[19] (2015). Metal-Related Antimicrobials Showcase Event.

[20] Katsikogianni M.G., Missirlis Y.F. (2004). Concise review of mechanisms of bacterial adhesion to biomaterials and of techniques used in estimating bacteria-material interactions. Eur. Cells Mater..

[21] Krishnan M., Seema S., Tiwari B., Sharma H.S., Londhe S., Arora V. (2015). Surface characterization of nickel titanium orthodontic arch wires. Med. J. Armed Forces India.

[22] Sridhar S., Abidi Z., Wilson T.G., Valderrama P., Wadhwani C., Palmer K., Rodrigues D.C. (2016). In vitro evaluation of the effects of multiple oral factors on dental implants surfaces. J. Oral Implantol..

[23] Verardi G., Cenci M.S., Maske T.T., Webber B., Santos L.R. (2016). Antiseptics and microcosm biofilm formation on titanium surfaces. Braz. Oral Res..

[24] Scheuerman T.R., Camper A.K., Hamilton M.A. (1998). Effects of substratum topography on bacterial adhesion. J. Colloid Interf. Sci..

[25] Edwards K.J., Rutenberg A.D. (2001). Microbial response to surface microtopography: The role of metabolism in localized mineral dissolution. Chem. Geol..

[26] Ojeil M., Jermann C., Holah J., Denyer S.P., Maillard J.Y. (2013). Evaluation of new in vitro efficacy test for antimicrobial surface activity reflecting UK hospital conditions. J. Hosp. Infect..

[27] Casey A.L., Adams D., Karpanen T.J., Lambert P.A., Cookson B.D., Nightingale P., Miruszenko L., Shillam R., Christian P., Elliott T.S.J. (2010). Role of copper in reducing hospital environment contamination. J. Hosp. Infect..

[28] Karpanen T.J., Casey A.L., Lambert P.A., Cookson B.D., Nightingale P., Miruszenko L., Elliott T.S.J. (2012). The antimicrobial efficacy of copper alloy furnishing in the clinical environment: A crossover study. Infect. Cont. Hosp. Epidemiol..

[29] O’Gorman J., Humphreys H. (2012). Application of copper to prevent and control infection. Where are we now?. J. Hosp. Infect..

[30] Robine E., Boulangé-Petermann L., Derangère D. (2002). Assessing bactericidal properties of materials: The case of metallic surfaces in contact with air. J. Microbiol. Methods.

[31] Cervantes H.I., Alvarez J.A., Munoz J.M., Arreguin V., Mosqueda J.L., Macias A.E. (2013). Antimicrobial activity of copper against organisms in aqueous solution: A case for copper-based water pipelines in hospitals?. Am. J. Infect. Control.

[32] Michels H.T., Noyce J.O., Keevil C.W. (2009). Effects of temperature and humidity on the efficacy of methicillin-resistant *Staphylococcus aureus* challenged antimicrobial materials containing silver and copper. Lett. Appl. Microbiol..

[33] Santo C.S., Taudte N., Nies D.H., Grass G. (2008). Contribution of copper ion resistance to survival of *Escherichia coli* on metallic copper surfaces. Appl. Environ. Microbiol..

[34] Mathews S., Hans M., Mucklich F., Solioz M. (2013). Contact Killing of Bacteria on Copper Is Suppressed if Bacterial-Metal Contact Is Prevented and Is Induced on Iron by Copper Ions. Appl. Environ. Microbiol..

[35] Molteni C., Abicht H.K., Solioz M. (2010). Killing of bacteria by copper surfaces involves dissolved copper. Appl. Environ. Microbiol..

[36] Lin B., Mu R., Yang L.F., Bian X.F. (2012). Antibacterial effect of metallic glasses. Chin. Sci. Bull..

[37] Jin K., Loffler J.F. (2005). Bulk metallic glass formation in Zr-Cu-Fe-Al alloys. Appl. Phys. Lett..

[38] Faúndez G., Troncoso M., Navarrete P., Figueroa G. (2004). Antimicrobial activity of copper surfaces against suspensions of *Salmonella enterica* and *Campylobacter jejuni*. BMC Microbiol..

[39] Nie Y., Kalapos C., Nie X., Murphy M., Hussein R., Zhang J. (2010). Superhydrophilicity and antibacterial property of a Cu-dotted oxide coating surface. Ann. Clin. Microbiol. Antimicrob..

[40] Stranak V., Wulff H., Ksirova P., Zietz C., Drache S., Cada M., Hubicka Z., Bader R., Tichy M., Helm C.A. (2014). Ionized vapor deposition of antimicrobial Ti-Cu films with controlled copper release. Thin Solid Films.

[41] Stranak V., Wulff H., Rebl H., Zietz C., Arndt K., Bogdanowicz R., Nebe B., Bader R., Podbielski A., Hubicka Z. (2011). Deposition of thin titanium-copper films with antimicrobial effect by advanced magnetron sputtering methods. Mater. Sci. Eng. C Mater..

[42] Wojcieszak D., Kaczmarek D., Antosiak A., Mazur M., Rybak Z., Rusak A., Osekowska M., Poniedzialek A., Gamian A., Szponar B. (2015). Influence of Cu-Ti thin film surface properties on antimicrobial activity and viability of living cells. Matr. Sci. Eng. C Mater..

[43] Arnell R.D., Kelly P.J. (1999). Recent advances in magnetron sputtering. Surf. Coat. Technol..

[44] Kelly P.J., Arnell R.D. (2000). Magnetron sputtering: A review of recent developments and applications. Vacuum.

[45] Chen H.W., Hsu K.C., Chan Y.C., Duh J.G., Lee J.W., Jang J.S.C., Chen G.J. (2014). Antimicrobial properties of Zr-Cu-Al-Ag thin film metallic glass. Thin Solid Films.

[46] Subramanian B. (2015). In vitro corrosion and biocompatibility screening of sputtered Ti_40_Cu_36_Pd_14_Zr_10_ thin film metallic glasses on steels. Mater. Sci. Eng. C Mater..

[47] Zhu L.B., Elguindi J., Rensing C., Ravishankar S. (2012). Antimicrobial activity of different copper alloy surfaces against copper resistant and sensitive *Salmonella enterica*. Food Microbiol..

[48] Chu Y.Y., Lin Y.S., Chang C.M., Liu J.K., Chen C.H., Huang J.C. (2014). Promising antimicrobial capability of thin film metallic glasses. Mater. Sci. Eng. C Mater..

[49] Ferreri I., Calderon V.S., Galindo R.E., Palacio C., Henriques M., Piedade A.P., Carvalho S. (2015). Silver activation on thin films of Ag-ZrCN coatings for antimicrobial activity. Mater. Sci. Eng. C Mater..

[50] Rebelo R., Manninen N.K., Fialho L., Henriques M., Carvalho S. (2016). Morphology and oxygen incorporation effect on antimicrobial activity of silver thin films. Appl. Surf. Sci..

[51] Wojcieszak D., Mazur M., Kaczmarek D., Mazur P., Szponar B., Domaradzki J., Kepinski L. (2016). Influence of the surface properties on bactericidal and fungicidal activity of magnetron sputtered Ti-Ag and Nb-Ag thin films. Mater. Sci. Eng. C Mater..

[52] Akhavan O. (2009). Lasting antibacterial activities of Ag-TiO_2_/Ag/a-TiO_2_ nanocomposite thin film photocatalysts under solar light irradiation. J. Colloid Interf. Sci..

[53] Foster H.A., Sheel D.W., Sheel P., Evans P., Varghese S., Rutschke N., Yates H.M. (2010). Antimicrobial activity of titania/silver and titania/copper films prepared by CVD. J. Photochem. Photobiol. A.

[54] Page K., Palgrave R.G., Parkin I.P., Wilson M., Savin S.L.P., Chadwick A.V. (2007). Titania and silver-titania composite films on glass-potent antimicrobial coatings. J. Mater. Chem..

[55] Yu B.Y., Leung K.M., Guo Q.Q., Lau W.M., Yang J. (2011). Synthesis of Ag-TiO_2_ composite nano thin film for antimicrobial application. Nanotechnology.

[56] Akhavan O., Ghaderi E. (2009). Bactericidal effects of Ag nanoparticles immobilized on surface of SiO_2_ thin film with high concentration. Curr. Appl. Phys..

[57] Hans M., Erbe A., Mathews S., Chen Y., Solioz M., Mucklich F. (2013). Role of Copper Oxides in Contact Killing of Bacteria. Langmuir.

[58] Hans M., Mathews S., Mucklich F., Solioz M. (2016). Physicochemical properties of copper important for its antibacterial activity and development of a unified model. Biointerphases.

[59] Dunlop P.S.M., Sheeran C.P., Byrne J.A., McMahon M.A.S., Boyle M.A., McGuigan K.G. (2010). Inactivation of clinically relevant pathogens by photocatalytic coatings. J. Photochem. Photobiol. A.

[60] Evans P., Sheel D.W. (2007). Photoactive and antibacterial TiO_2_ thin films on stainless steel. Surf. Coat. Technol..

[61] Yates H.M., Brook L.A., Ditta I.B., Evans P., Foster H.A., Sheel D.W., Steele A. (2008). Photo-induced self-cleaning and biocidal behaviour of titania and copper oxide multilayers. J. Photochem. Photobiol. A.

[62] Reddy M.P., Venugopal A., Subrahmanyam M. (2007). Hydroxyapatite-supported Ag-TiO2 as Escherichia coli disinfection photocatalyst. Water Res..

[63] Dancer S.J. (2009). The role of environmental cleaning in the control of hospital-acquired infection. J. Hosp. Infect..

[64] Dancer S.J. (2011). Hospital cleaning in the 21st century. Eur. J. Clin. Microbiol. Infect. Dis..

[65] Dancer S.J. (2004). How do we assess hospital cleaning? A proposal for microbiological standards for surface hygiene in hospitals. J. Hosp. Infect..

[66] Gillespie E., Wright P.L., Snook K., Ryan S., Vandergraaf S., Abernethy M., Lovegrove A. (2015). The role of ultraviolet marker assessments in demonstrating cleaning efficacy. Am. J. Infect. Control.

[67] Griffith C.J., Cooper R.A., Gilmore J., Davies C., Lewis M. (2000). An evaluation of hospital cleaning regimes and standards. J. Hosp. Infect..

[68] Malik R.E., Cooper R.A., Griffith C.J. (2003). Use of audit tools to evaluate the efficacy of cleaning systems in hospitals. Am. J. Infect. Control.

[69] Murphy C.L., Macbeth D.A., Derrington P., Gerrard J., Faloon J., Kenway K., Lavender S., Leonard S., Orr A., Tobin D. (2012). An assessment of high touch object cleaning thoroughness using a fluorescent marker in two Australian hospitals. Healthc. Infect..

[70] Airey P., Verran J. (2007). Potential use of copper as a hygienic surface; problems associated with cumulative soiling and cleaning. J. Hosp. Infect..

[71] Copper Development Association. http://copperalliance.org.uk.

[72] Lalitha A., Ramesh S., Rajeswari S. (2005). Surface protection of copper in acid medium by azoles and surfactants. Electrochim. Acta.

[73] Rutala W.A., Weber D.J. (1997). Uses of inorganic hypochlorite (bleach) in health-care facilities. Clin. Microbiol. Rev..

[74] Lavorgna M., Russo C., D’Abrosca B., Parrella A., Isidori M. (2016). Toxicity and genotoxicity of the quaternary ammonium compound benzalkonium chloride (BAC) using *Daphnia magna* and *Ceriodaphnia dubia* as model systems. Environ. Pollut..

[75] Wolkoff P., Schneider T., Kildeso J., Degerth R., Jaroszewski M., Schunk H. (1998). Risk in cleaning: Chemical and physical exposure. Sci. Total Environ..

[76] Cadnum J.L., Mana T.S.C., Jencson A., Thota P., Kundrapu S., Donskey C.J. (2015). Effectiveness of a hydrogen peroxide spray for decontamination of soft surfaces in hospitals. Am. J. Infect. Control.

[77] Flores M.J., Brandi R.J., Cassano A.E., Labas M.D. (2012). Chemical disinfection with H_2_O_2_—The proposal of a reaction kinetic model. Chem. Eng. J..

[78] Labas M.D., Zalazar C.S., Brandi R.J., Cassano A.E. (2008). Reaction kinetics of bacteria disinfection employing hydrogen peroxide. Biochem. Eng. J..

[79] Bartels M.D., Kristoffersen K., Slotsbjerg T., Rohde S.M., Lundgren B., Westh H. (2008). Environmental meticillin-resistant *Staphylococcus aureus* (MRSA) disinfection using dry-mist-generated hydrogen peroxide. J. Hosp. Infect..

[80] Piskin N., Celebi G., Kulah C., Mengeloglu Z., Yumusak M. (2011). Activity of a dry mist-generated hydrogen peroxide disinfection system against methicillin-resistant *Staphylococcus aureus* and *Acinetobacter baumannii*. Am. J. Infect. Control.

[81] Andersen B.M., Rasch M., Hochlin K., Jensen F.H., Wismar P., Fredriksen J.E. (2006). Decontamination of rooms, medical equipment and ambulances using an aerosol of hydrogen peroxide disinfectant. J. Hosp. Infect..

[82] Fu T.Y., Gent P., Kumar V. (2012). Efficacy, efficiency and safety aspects of hydrogen peroxide vapour and aerosolized hydrogen peroxide room disinfection systems. J. Hosp. Infect..

[83] Thomas E.L., Milligan T.W., Joyner R.E., Jefferson M.M. (1994). Antibacterial activity of hydrogen-peroxide and the lactoperoxidase-hydrogen peroxide-thiocyanate system against oral streptococci. Infect. Immun..

[84] McDonnell G. (2009). The Use of Hydrogen Peroxide for Disinfection and Sterilization Applications. PATAI’S Chemistry of Functional Groups.

[85] DeNardis D., Rosales-Yeomans D., Borucki L., Philipossian A. (2006). Characterization of copper-hydrogen peroxide film growth kinetics. Thin Solid Films.

[86] Barillo D.J., Marx D.E. (2014). Silver in medicine: A brief history BC 335 to present. Burns.

[87] Kim J.S., Kuk E., Yu K.N., Kim J.H., Park S.J., Lee H.J., Kim S.H., Park Y.K., Park Y.H., Hwang C.Y. (2007). Antimicrobial effects of silver nanoparticles. Nanomedicine.

[88] Armon R., Laot N., Lev O., Shuval H., Fattal B. (2000). Controlling biofilm formation by hydrogen peroxide and silver combined disinfectant. Water Sci. Technol..

[89] Pasquet J., Chevalier Y., Pelletier J., Couval E., Bouvier D., Bolzinger M.-A. (2014). The contribution of zinc ions to the antimicrobial activity of zinc oxide. Colloids Surf. A Physicochem. Eng. Asp..

[90] Ercan D., Cossu A., Nitin N., Tikekar R.V. (2016). Synergistic interaction of ultraviolet light and zinc oxide photosensitizer for enhanced microbial inactivation in simulated wash-water. Innov. Food Sci. Emerg. Technol..

[91] Wöll C. (2007). The chemistry and physics of zinc oxide surfaces. Prog. Surf. Sci..

[92] Sawai J., Shoji S., Igarashi H., Hashimoto A., Kokugan T., Shimizu M., Kojima H. (1998). Hydrogen peroxide as an antibacterial factor in zinc oxide powder slurry. J. Ferment. Bioeng..

[93] Zhang L., Li Y., Liu X., Zhao L., Ding Y., Povey M., Cang D. (2013). The properties of ZnO nanofluids and the role of H_2_O_2_ in the disinfection activity against *Escherichia coli*. Water Res..

[94] Roberge P.R. (2012). Engineering Materials: Selection and Design Considerations. Handbook of Corrosion Engineering, Second Edition.

[95] Montes J.C., Hamdani F., Creus J., Touzain S., Correc O. (2014). Impact of chlorinated disinfection on copper corrosion in hot water systems. Appl. Surf. Sci..

[96] Sierra M., Sanhueza A., Alcántara R., Sánchez G. (2013). Antimicrobial evaluation of copper sulfate (II) on strains of *Enterococcus faecalis*. In vitro study. J. Oral Res..

[97] Rai M., Yadav A., Gade A. (2009). Silver nanoparticles as a new generation of antimicrobials. Biotechnol. Adv..

[98] Hernández-Sierra J.F., Ruiz F., Cruz Pena D.C., Martínez-Gutiérrez F., Martínez A.E., de Jesús Pozos Guillén A., Tapia-Pérez H., Martínez Castañón G. (2008). The antimicrobial sensitivity of *Streptococcus mutans* to nanoparticles of silver, zinc oxide, and gold. Nanomedicine.

[99] Hsu M.S., Wu M.Y., Huang Y.T., Liao C.H. (2016). Efficacy of chlorine dioxide disinfection to non-fermentative Gram-negative bacilli and non-tuberculous mycobacteria in a hospital water system. J. Hosp. Infect..

[100] Cooper I.R., Hanlon G.W. (2010). Resistance of *Legionella pneumophila* serotype 1 biofilms to chlorine-based disinfection. J. Hosp. Infect..

[101] Kim J., Pitts B., Stewart P.S., Camper A., Yoon J. (2008). Comparison of the Antimicrobial Effects of Chlorine, Silver Ion, and Tobramycin on Biofilm. Antimicrob. Agents Chemother..

[102] Landeen L.K., Yahya M.T., Gerba C.P. (1989). Efficacy of copper and silver ions and reduced levels of free chlorine in inactivation of *Legionella pneumophila*. Appl. Environ. Microbiol..

[103] McDonnell G., Russell A.D. (1999). Antiseptics and disinfectants: Activity, action, and resistance. Clin. Microbiol. Rev..

[104] Kampf G., Rudolf M., Labadie J.C., Barrett S.P. (2002). Spectrum of antimicrobial activity and user acceptability of the hand disinfectant agent Sterillium Gel. J. Hosp. Infect..

[105] Brady M.J., Lisay C.M., Yurkovetskiy A.V., Sawan S.P. (2003). Persistent silver disinfectant for the environmental control of pathogenic bacteria. Am. J. Infect. Control.

[106] Guthery E., Seal L.A., Anderson E.L. (2005). Zinc pyrithione in alcohol-based products for skin antisepsis: Persistence of antimicrobial effects. Am. J. Infect. Control.

[107] Seal L.A., Rizer R.L., Maas-Irslinger R. (2005). A unique water optional health care personnel handwash provides antimicrobial persistence and residual effects while decreasing the need for additional products. Am. J. Infect. Control.

[108] Hall T.J., Wren M.W., Jeanes A., Gant V.A. (2009). A comparison of the antibacterial efficacy and cytotoxicity to cultured human skin cells of 7 commercial hand rubs and Xgel, a new copper-based biocidal hand rub. Am. J. Infect. Control.

[109] Zaragoza Dörwald F. (2012). Quaternary Ammonium Salts. Lead Optimization for Medicinal Chemists.

[110] Nakagawa Y., Hayashi H., Tawaratani T., Kourai H., Horie T., Shibasaki I. (1984). Disinfection of water with quaternary ammonium salts insolubilized on a porous glass surface. Appl. Environ. Microbiol..

[111] Shirai A., Aihara M., Takahashi A., Maseda H., Omasa T. (2014). Synergistic antimicrobial activity based on the combined use of a gemini-quaternary ammonium compound and ultraviolet-A light. J. Photochem. Photobiol. B Biol..

[112] Francavilla C., Low E., Nair S., Kim B., Shiau T.P., Debabov D., Celeri C., Alvarez N., Houchin A., Xu P. (2009). Quaternary ammonium *N*,*N*-dichloroamines as topical, antimicrobial agents. Bioorganic Med. Chem. Lett..

[113] Ioannou C.J., Hanlon G.W., Denyer S.P. (2007). Action of Disinfectant Quaternary Ammonium Compounds against *Staphylococcus aureus*. Antimicrob. Agents Chemother..

[114] Makvandi P., Ghaemy M., Mohseni M. (2016). Synthesis and characterization of photo-curable bis-quaternary ammonium dimethacrylate with antimicrobial activity for dental restoration materials. Eur. Polym. J..

[115] Harrison J.J., Turner R.J., Joo D.A., Stan M.A., Chan C.S., Allan N.D., Vrionis H.A., Olson M.E., Ceri H. (2008). Copper and Quaternary Ammonium Cations Exert Synergistic Bactericidal and Antibiofilm Activity against *Pseudomonas aeruginosa*. Antimicrob. Agents Chemother..

[116] Hegazy M.A., Nazeer A.A., Shalabi K. (2015). Electrochemical studies on the inhibition behavior of copper corrosion in pickling acid using quaternary ammonium salts. J. Mol. Liq..

[117] Collins K.J. (1957). The corrosion of metal by palmar sweat. Br. J. Ind. Med..

[118] Bond J.W. (2008). Visualization of latent fingerprint corrosion of metallic surfaces. J. Forensic Sci..

[119] Fredj N., Kolar J.S., Prichard D.M., Burleigh T.D. (2013). Study of relative color stability and corrosion resistance of commercial copper alloys exposed to hand contact and synthetic hand sweat. Corros. Sci..

[120] Tu M.-E., Wu Y.-H. (2011). Multiple allergies to metal alloys. Dermatol. Sin..

[121] Goodwin F.E., Cottis B., Graham M., Lindsay R., Lyon S., Richardson T., Scantlebury D., Stott H. (2010). Corrosion of Zinc and its Alloys. Shreir’s Corrosion.

[122] Lyon S.B., Cottis B., Graham M., Lindsay R., Lyon S., Richardson T., Scantlebury D., Stott H. (2010). Corrosion of Noble Metals. Shreir’s Corrosion.

[123] Tuck C.D.S., Powell C.A., Nuttall J., Cottis B., Graham M., Lindsay R., Lyon S., Richardson T., Scantlebury D., Stott H. (2010). Corrosion of Copper and its Alloys. Shreir’s Corrosion.

[124] Daniels S.L., Sprunger P.T., Kizilkaya O., Lytle D.A., Garno J.C. (2013). Nanoscale surface characterization of aqueous copper corrosion: Effects of immersion interval and orthophosphate concentration. Appl. Surf. Sci..

[125] Mann E.E., Manna D., Mettetal M.R., May R.M., Dannemiller E.M., Chung K.K., Brennan A.B., Reddy S.T. (2014). Surface micropattern limits bacterial contamination. Antimicrob. Resist. Infect. Control.

